# SMARCA4-Deficient Undifferentiated Esophageal Carcinoma: A Clinical Case Series and Literature Review

**DOI:** 10.1007/s12029-024-01060-4

**Published:** 2024-04-24

**Authors:** Faris Shweikeh, Gordon Hong, Jacob Walter, Matthew Hoscheit, Anthony Lembo, Mohamad Mouchli, Jason Lane

**Affiliations:** 1 Department of Internal Medicine, Cleveland Clinic Akron General, 1 Akron General Ave., Akron, OH 44307 USA; 2 Department of Internal Medicine, University Hospitals Cleveland Medical Center, Cleveland, OH USA; 3 College of Medicine, Northeast Ohio Medical University, Rootstown, OH USA; 4 Department of Gastroenterology, Hepatology and Nutrition, Digestive Disease and Surgery Institute, Cleveland Clinic Foundation, Cleveland, OH USA; 5 Department of Pathology, Cleveland Clinic Akron General, Akron, OH USA

**Keywords:** Esophageal cancer, Undifferentiated carcinoma, Chemoradiation, SMARCA genes, Histopathology

## Abstract

**Purpose:**

Undifferentiated carcinoma of the esophagus (UEC) is a rare malignancy. Deficiency in SMARCA genes, critical for chromatin regulation, has been observed in cases of UEC. Research in UEC is sparse, however, and we present a case series along with a comprehensive review of the literature.

**Case Series:**

Case 1 is a 49-year-old female with abdominal pain and dysphagia and esophagogastroduodenoscopy (EGD) showing a friable mass at the gastroesophageal (GE) junction. Biopsies showed a poorly differentiated neoplasm and immunohistochemistry showed loss for SMARCA4. With metastatic disease, she agreed to undergo palliative chemotherapy and radiation, passing away at 4 months. Case 2 is an 88-year-old male with dysphagia, nausea, vomiting, and distal esophageal mass with biopsy showing a malignancy with loss of SMARCA4 expression. Due to extensive metastases, he was counseled on hospice care. Case 3 is a 53-year-old male with extensive alcohol and smoking history presenting with hematemesis, passing away shortly. Posthumous histopathology consistent with undifferentiated SMARCA4-deficient carcinoma of the esophagus. Results of the literature review indicate a predilection towards males (75.0%) and a variable age range (39–88 years). Majority (76.2%) reported with a distal esophagus location. Metastatic disease was common at initial presentation. Median survival was 2.60 months. Some were managed with chemotherapy and radiation.

**Conclusions:**

Research in SMARCA-deficient UEC is very limited. It is more common in men, age is variable, and associated with Barret’s esophagus. Further research is necessary to better understand it and to establish treatment guidelines; however, it is clear that SMARCA4-deficient UEC carries a significantly poor prognosis.

## Introduction

As of 2020, esophageal cancer (EC) was reported to rank seventh in incidence and sixth in overall mortality among cancers worldwide [1]. Esophageal squamous cell carcinoma (ESCC) and adenocarcinoma (EAC) are the two main types with 80% of cases being ESCC, which can develop through all regions of the esophagus [2]. It is estimated that the incidence of EAC will continue to increase in countries of higher income, while the incidence of ESCC is projected to decrease in predilected countries [2]. Tobacco smoke and alcohol are well-known risk factors for ESCC, while gastroesophageal reflux disease (GERD) and Barrett’s esophagus are the greatest risk factors for EAC. Management of esophageal cancer depends on stage and can include endoscopic resection, stent placement, esophagectomy, radiation therapy, and chemotherapy. Prognosis and survival for patients with esophageal cancer, however, remains poor [3]. Undifferentiated carcinoma of the esophagus is a rare subset of esophageal cancer reported mainly in case reports and case series and was at least initially associated with an especially aggressive course and worse outcomes [4]. Research into SMARCA4 deficient undifferentiated esophageal carcinoma (UEC) is ongoing, and here, we present a series of three cases and briefly review some of the most pertinent literature to enhance understanding of its clinical presentation, histopathological findings, treatment challenges, and outcomes.

## Case Series

### Case 1

The patient is a 49-year-old female with a remote history of tobacco abuse, endometrial cancer, status post hysterectomy, and GERD who presented to the ED with a 3-week history of abdominal pain and dysphagia but left before being evaluated. Later that month she had an appointment with outpatient gastroenterology. Physical exam showed a well-nourished female with a BMI of 42.9 kg/m^2^, abdominal distention, and mild diffuse abdominal tenderness without rebound tenderness. Laboratory findings, including complete metabolic panel and complete blood count, showed slight leukocytosis at 12.0 k/µL, normal electrolytes, and elevated c-reactive protein (CRP) at 4.3 mg/dL. An esophagogastroduodenoscopy (EGD) showed a friable mass at the GE junction within an area of possible Barrett’s esophagus. Biopsies showed necrotic debris and Candida pseudohyphae.

**Fig. 1:**
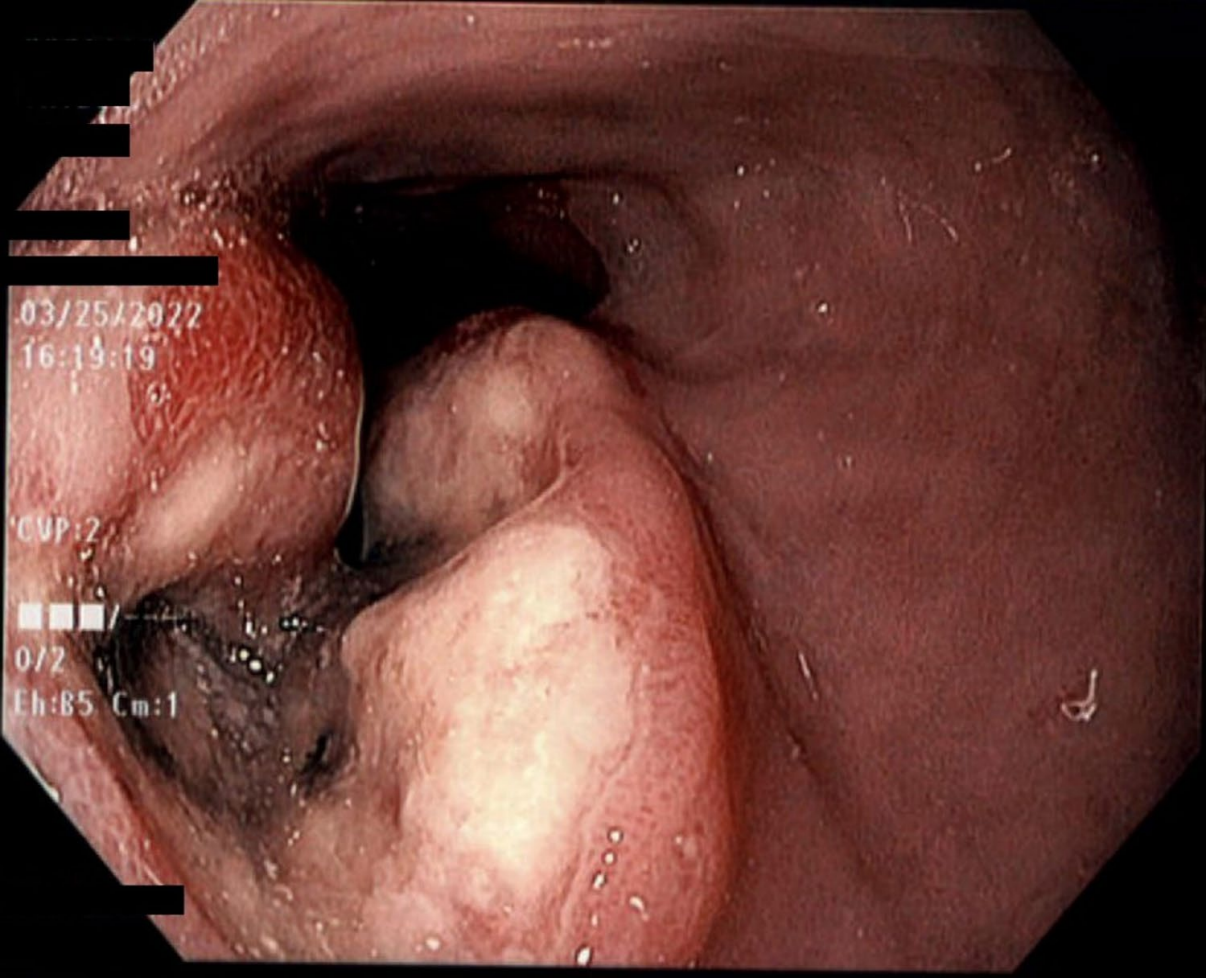
Endoscopy from Case 1 showed a necrotic friable, hard mass extending from 30 to 38 cm. Salmon colored mucosa noted in the background in combination with the mass suggested malignancy in the background of Barrett’s esophagus

She followed up with oncology and had a repeat EGD with biopsies taken (Fig. 1), which this time showed a poorly differentiated malignant neoplasm with extensive necrosis. The focal intact neoplasm demonstrated large cells with irregular vesicular nuclei, often with a prominent nucleolus, and moderate eosinophilic cytoplasm. Immunohistochemistry for SMARCA4 showed loss of nuclear expression in the neoplastic cells. Notably, intestinal metaplasia was noted in one of the biopsy fragments. Additionally, immunohistochemistry (IHC) for HER2 (ERBB2) was equivocal and reflex FISH testing for HER2 (ERBB2) showed a non-amplified result. Immunohistochemistry for PD-L1 showed a combined positive score (CPS) of < 1. Mismatch repair (MMR) testing was not performed in this case.

An initial plan was made for neoadjuvant chemotherapy and radiation therapy followed by surgery with curative intent. She began having worsening dysphagia and severe epigastric pain and was admitted. A PET/CT was obtained which showed possible metastatic disease in the left axillary lymph node and right gluteus muscle. Imaging-guided biopsies of both lesions were obtained and showed findings that were histologically identical to her esophageal carcinoma. Following the finding of metastatic disease, she agreed to undergo chemotherapy and radiation therapy with palliative intent. Systemic chemotherapy with FOLFOX was initiated.

She had only received one cycle of chemotherapy before she started having complications including severe pain and lymphadenopathy compressing her port site, interfering with the remainder of her treatment. She did receive a few palliative radiation treatments to help with this. A few weeks later she was hospitalized for pneumonia and sepsis, progressing to respiratory failure for which she was intubated. Discussions were held with her family before extubation, and the patient passed away shortly thereafter, approximately 4 months after diagnosis.

### Case 2

The patient is an 88-year-old male with a past medical history of hypertension, hyperlipidemia, congestive heart failure, atrial fibrillation, chronic obstructive pulmonary disease, diabetes mellitus, hypothyroidism, remote colon cancer, and GERD, who presented to the ED with worsening shortness of breath. He was worked up and hospitalized for pneumonia at the same time that he was also being evaluated for ongoing dysphagia to both solids and liquids, nausea, and vomiting. A chest X-ray showed a large hiatal hernia and an EGD was performed. A large friable mass was noted in the distal esophagus, and biopsies were taken.

**Fig. 2:**
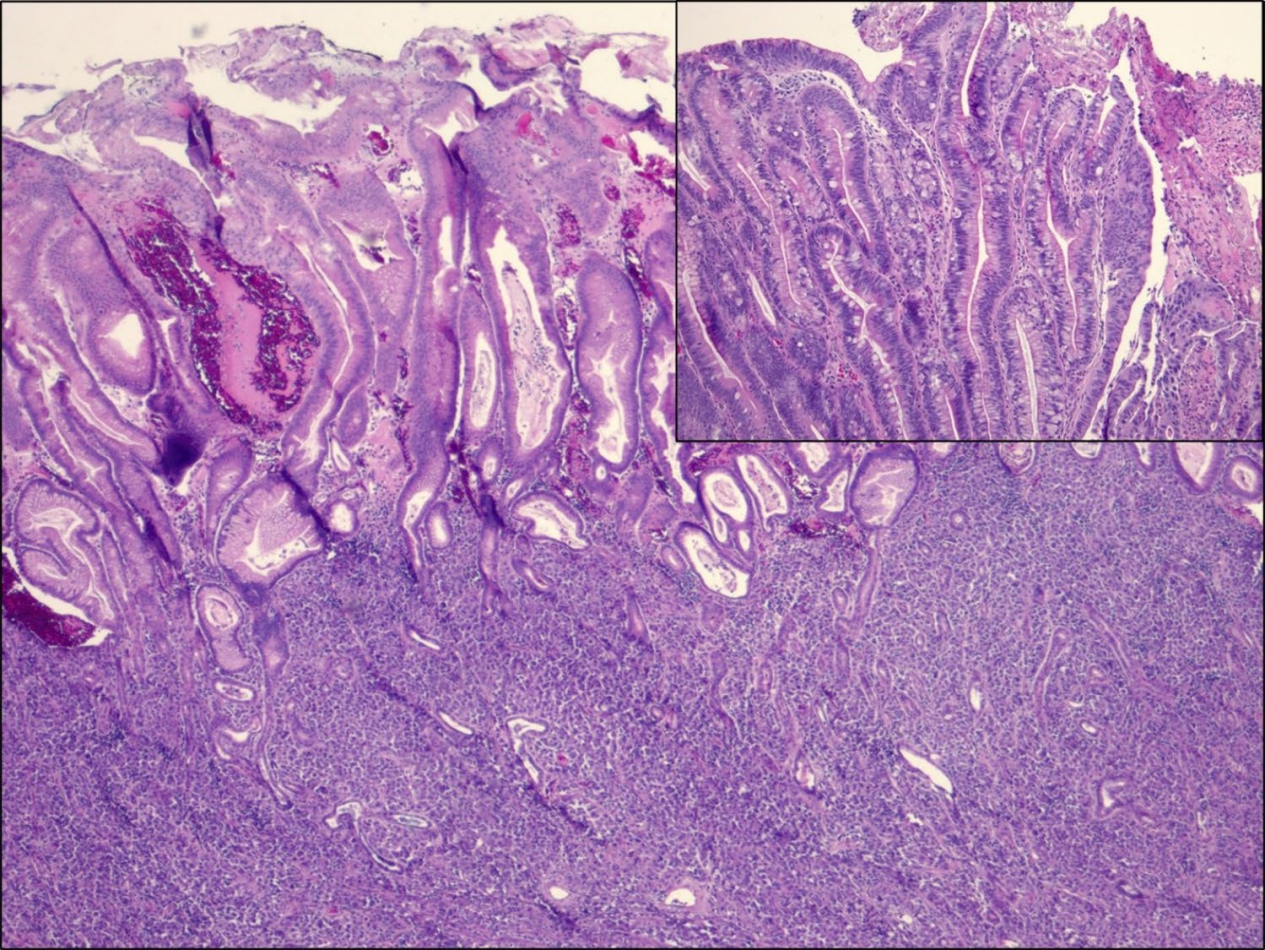
H&E-stained sections of tumor from Case 2 showed an invasive high-grade epithelioid malignancy with sheet-like pattern of growth infiltrating below benign glandular epithelium (×40 total magnification). Separate biopsy fragments demonstrated Barrett’s esophagus with glandular dysplasia (inset, ×100)

**Fig. 3:**
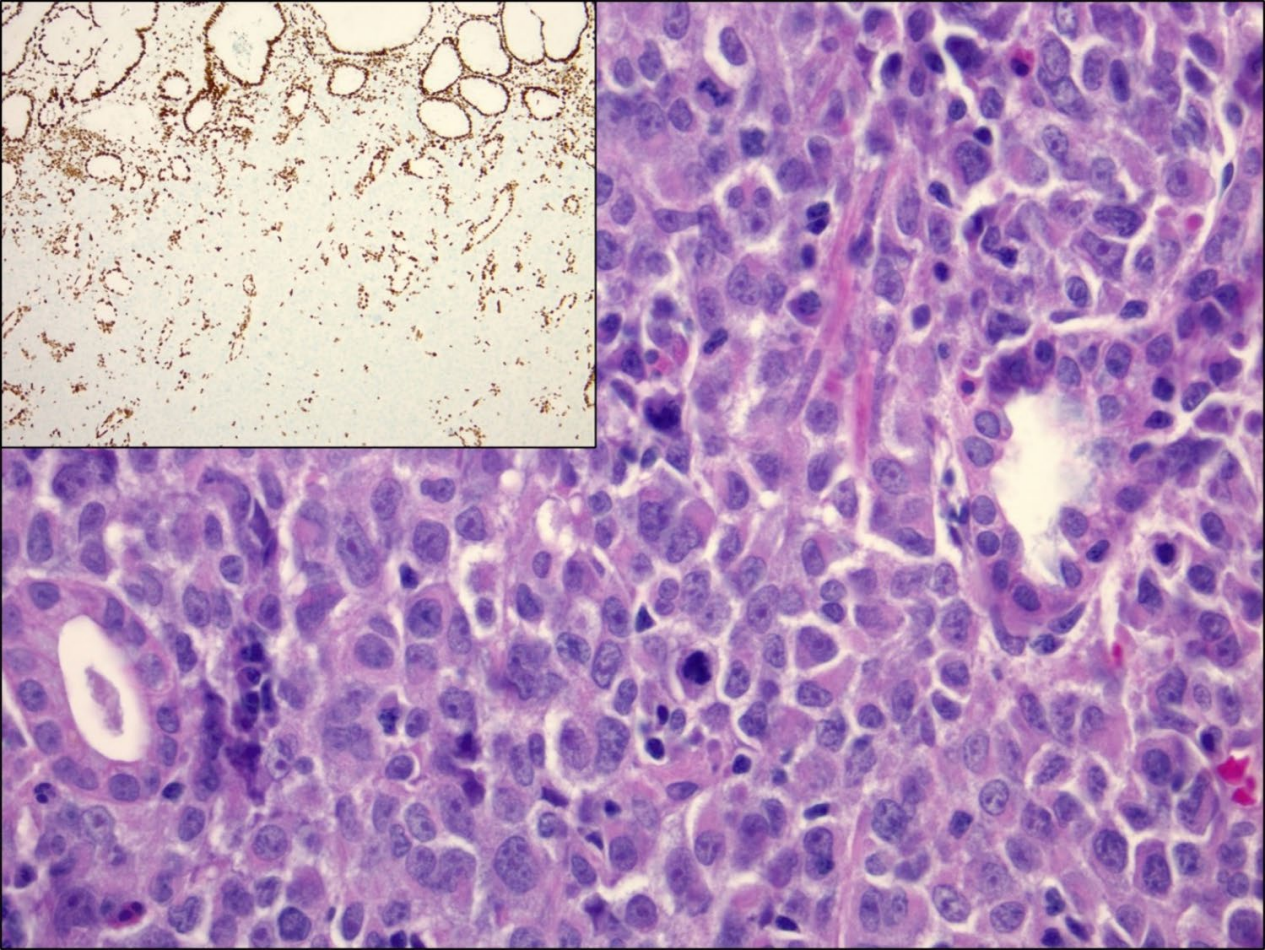
High magnification (×400) H&E-stained section from Case 2 showed discohesive cells with moderate amounts of eosinophilic cytoplasm and focal features suggestive of rhabdoid morphology (center of image). The tumor nuclei were enlarged and somewhat monomorphic with prominent nucleoli and mitotic activity was brisk. SMARCA4 immunohistochemistry (inset, ×100) showed loss of nuclear expression in the tumor cells while retained within the benign glandular epithelium and inflammatory cells which served as a positive internal control

Histopathology showed an invasive high-grade epithelioid malignancy with a sheet-like pattern of somewhat discohesive cells in a background of Barrett’s esophagus with high-grade glandular dysplasia (Fig. 2). The tumor cells showed moderate amounts of eosinophilic cytoplasm with focal features suggestive of rhabdoid morphology. SMARCA4 immunohistochemistry showed loss of nuclear expression in the tumor cells (Fig. 3). Overall, the pathologic findings were consistent with a poorly differentiated carcinoma with SMARCA4 deficiency. HER2 testing by immunohistochemistry was positive (score 3+). Mismatch repair testing by IHC showed a proficient result. Immunohistochemistry for PD-L1 showed a CPS of < 1. A later CT showed possible metastatic disease in the liver, which was biopsied and showed identical histological findings. A pleural effusion was sampled and found to also consist of malignant cells. His clinical course was complicated by admission to the intensive care unit for worsening respiratory status. Due to stage IV disease, he was not considered a good surgical candidate, and after multidisciplinary team discussion and consideration of clinical and performance status, patient opted to avoid chemotherapy and radiation therapy, and he was counseled on hospice care.

He was eventually discharged to home on total parenteral nutrition and comfort care measures, where he passed away shortly thereafter, approximately 1 month after diagnosis.

### Case 3

The patient is a 53-year-old male with a history of daily alcohol averaging 3–4 drinks per day and 15 pack year history of smoking who presented outpatient with 2-month history of fatigue, abdominal pain, vomiting, decreased appetite, and roughly 20 lbs weight loss**.** Right upper quadrant ultrasound showed multiple discrete lesions in the liver concerning metastatic disease. CT showed severe wall thickening of the distal esophagus suspicious for esophageal carcinoma as well as innumerable liver nodules and calcified lung nodules both suspicious for metastases. An EGD was performed and biopsies were taken. A few days later he presented to the ED with severe abdominal pain and hematemesis. He was hospitalized and passed away the next day due to respiratory failure.

Posthumous histopathological exam of the esophageal biopsies was consistent with undifferentiated SMARCA4-deficient carcinoma. Because the patient was deceased at the time the pathology report was finalized, ancillary testing for HER2, PD-L1, and MMR was not performed.

A meticulous search of the current literature on this topic yielded the articles summarized in Table 1. Results of the literature review indicate males made up most cases (75.0%), with a variable age range (39–88 years). Majority of patients (76.2%) had lesions in the distal esophagus or GEJ. EGD and CT typically showed a friable or ulcerating and/or esophageal thickening. Liver metastasis was common on presentation. Average survival following diagnosis is dismal at 2.35 months. Some patients were offered chemotherapy and radiation, although many passed away shortly after diagnosis.

**Table 1:** Previous reported cases of SMARCA4/ SMARCA2-deficient undifferentiated esophageal carcinoma

**Authors**	***n***	**Patient age (years)**	**Patient sex**	**Location within esophagus (distance from incisors)**	**Stage/sites of metastasis**	**Treatment**	**Outcome**	**Special notes**
Current series	3	Case 1: 49	F	GEJ	LN, muscle	Chemo, RT	Death at 4 mos.	NA
		Case 2: 88	M	Distal	Liver, lung	None	Death at 1 mos.	
		Case 3: 53	M	Distal	Liver, lung	None	Death at 0 mos.	
Cui et al. [6]	4	Case 1: 68	F	Mid-distal (30–40 cm)	Stage IVB, liver	EGD w/stent	Death at 72 days	NA
		Case 2: 47	M	Distal (37 cm)	Stage IVB, liver	Clinical trial, chemo, panitumumab	Death at 78 days	
		Case 3: 45	M	Distal	Stage III	FOLFOX, carboplatin, paclitaxel, RT, esophagectomy	Death at 8 mos.	
		Case 4: 55	M	30–37 cm	Stage III	FOLFOX	Death at 3 mos.	
Neil et al. [13]	8	NR	NR	NR	NR	NR	NR	Pathological study of both esophageal and gastric carcinoma
Ahmed et al. [5]	2	Case 1: 39	M	Distal	liver	None	Death at 1.5 mos.	NA
		Case 2: 64	M	Distal	liver	None	Death at 3 mos.	
Horton et al. [4]	14	Mean: 73.1 ± 7.2	10 M 4 F	GEJ: 2 Distal:5 Mid: 4 NR: 4	NR	NR	Limited follow-up available: 3 with death at 0.6, 2, and 7 mos.	Limited clinical data and follow-up
Agaimy et al. [9]	1	54	M	GEJ	Liver, lung, mediastinal	Neoadjuvant chemo, resection, palliative chemo	Alive at 1 year	Series of 13 cases of UC of GI tract

*M* male, *F* female, *EGD* esophagogastroduodenoscopy, *chemo* chemotherapy, *FOLFOX* folinic acid, fluorouracil, and oxaliplatin, *RT* radiotherapy, *NA* not applicable, *NR* not reported, *GEJ* gastroesophageal junction, *UC* undifferentiated carcinoma

## Discussion

Undifferentiated carcinoma of the esophagus with SMARCA4 and/or SMARCA2 deficiency is a rare tumor associated with a very poor prognosis [4]. Two out of the three cases we presented herein were males, coinciding with what previous authors have suggested—a predilection towards male sex [4–7]. As the age range previously reported varies, we similarly report a variable age range with a patient as young as 47 years. In terms of localization, the cancer occurred in the distal esophagus or GE junction with our cases, in line with what others have reported previously [4–6]. We find that all cases either initially presented with or shortly thereafter developed metastatic disease. The range of survival after presentation or diagnosis was between 2 and 4 months, though slighter lower, and is still comparable to what has been reported in the past [4–6]. As a result, treatment outcomes were very dismal in all three of our cases.

Undifferentiated esophageal carcinoma is challenging to diagnose given the rarity of this disease, lack of established diagnostic criteria, and absence of microscopic features typically used to characterize other malignancies. Deficiency in SMARCA genes, critical for chromatin regulation, has been observed in cases of undifferentiated neoplasms at other anatomic sites [8]. SMARCA4 and SMARCA2 are two important genes in the field of cancer genetics [4]. These genes encode subunits of the SWI/SNF chromatin remodeling complex, and loss-of-function mutations in these genes often act as the molecular basis for oncogenesis [4]; the protein products of the mutated genes form a SWI/SNF complex act together as a functional unit [9]. Mutations in these genes have been shown to play a key role in the development of certain malignancies, specifically rhabdoid tumors, small cell carcinoma of the ovary, hypercalcemic type, and non-small cell lung cancer [4, 8]. Importantly, SMARCA4 mutations typically signal a bleak prognosis and difficult clinical course [8, 10, 11].

One of the first reports of UEC tumors in the esophagus was reported in 2015 by Singhi et al., which reported male predominance, mean age of 65.5 years, 75% association with Barrett’s, and all patients dying at last follow-up [7]. Details with regards to SMARCA4 deficiency were not reported. The following year, Agaimy and colleagues reported on cases throughout the GI tract with one in the esophagus having SMARCA2 loss [9]. In the largest case series to date, Horton et al. describe the pathologic findings and clinical features of UEC with SMARCA4 and/or SMARCA2 deficiency in a case series of 14 patients [4]. In their report, the majority of cases were found among men (10/14), age at diagnosis ranged from 63 to 86 years, and half of cases originated from the distal esophagus or gastroesophageal junction. Patient follow-up in their series is significantly limited; however, they report known mortality of 3 patients from their cohort at 0.6, 2, and 7 months after diagnosis. The authors note the tumor cells of this malignancy as having enlarged nuclei, prominent nucleoli, and moderate eosinophilic cytoplasm, with 9 of 14 cases showing extensive necrosis, and 4 of 14 cases showing cells with rhabdoid morphology. Additionally, 8 of 14 cases showed adjacent intestinal epithelium with goblet cells, suggesting an association with Barrett’s esophagus, a precursor lesion that notably is also more likely to be found in males.

Cui et al. report a case series of four patients with SMARCA4-deficient UEC and similarly, the majority of these cases were male (3/4) [6]. This cohort is notably younger, at ages 45, 47, 55, and 68. At the time of diagnosis, two of four of this cohort were found to have stage III disease with locally advanced cancer whereas the other half were found to be at stage IVB, both with metastatic disease to the liver. Among the patients with stage III disease, one was treated with chemotherapy followed by esophagectomy while the other received chemotherapy alone. Both patients however were found to have progression to metastatic disease and eventual death less than 1 year after diagnosis. Patients with metastatic disease at diagnosis in this cohort underwent palliative measures, with death observed at 72 days and 78 days after diagnosis. Regarding risk factors, all patients denied smoking history and only one of four patients reported notable alcohol consumption at 5–10 standard drinks per week. Ahmed et al. also report a small case series of two male patients with SMARCA4-deficient UEC [5]. Interestingly, one of these patients was particularly young at 39 years of age while the other was 64 years old. Similarly, both patients presented with stage IVB disease with hepatic metastasis. Mortality in these patients was observed at 1.5 months and 3 months after initial presentation.

More recently, molecular characterization of these tumors on a large number of cases was reported in two studies [12, 13]. One specifically looked at all the GE junction carcinomas at a single institution which had molecular analysis performed and identified which ones had SMARCA4 mutation [13]. Then, from that subset they identified by histology which cases were undifferentiated versus well, moderate, or poorly differentiated. Subsequently, they identified the specific types of mutations occurring in the SMARCA4 gene (i.e., protein truncating versus missense) and correlated that with protein expression. Eight of 12 (75%) of cancers with protein-truncating SMARCA4 variants demonstrated a loss of SMARCA4 protein expression by IHC, whereas none of the seven cancers with pathogenic missense SMARCA4 variants demonstrated a loss of SMARCA4 protein expression. Although a more detailed clinical description of these individual cases was not provided in the article their survival analyses showed that patient outcome was associated with stage. Interestingly, not all carcinomas with SMARCA4 truncating variants demonstrated an undifferentiated phenotype, and a range of morphologic features were observed which did trend towards higher histologic grade: 37% were moderately differentiated, 53% were poorly differentiated, and 11% were undifferentiated. While the authors propose SMARCA4-deficient gastroesophageal carcinomas may not represent a unique tumor subtype, a more complete understanding of the full spectrum of SMARCA4 mutations will help define the patient population that may benefit from drug development. Further, the authors report that HER2 (ERBB2) gene amplification was observed in 5 (12%) of 42 carcinomas with pathogenic SMARCA4 mutation. Since we identified a single positive HER2 result in our series of three patients, HER2 testing would appear to be useful to identify patients that may benefit from trastuzumab (Herceptin) therapy.

Currently, no systemic chemotherapy regimen has proven to be effective in treating SWI/SNF-deficient malignancies, which include SMARCA2/SMARCA4-deficient tumors such as the cases described in this report. Thus, there is clearly a clinical need for effective treatment. The first step however is to accurately diagnose these tumors. Widespread availability and utilization of SWI/SNF immunohistochemistry in surgical pathology practices would be a good first step in screening for these tumors. However, as highlighted earlier, protein-truncating variants demonstrate a loss of SMARCA4 protein expression by IHC, whereas pathogenic missense SMARCA4 variants typically show retained expression thus masking the underlying mutation. Molecular testing would be a useful ancillary test to identify both types of mutations more accurately. Few commercial as well as academic laboratories have begun to include SWI/SNF-related genes (e.g., SMARCA4, SMARCB1) in their next-generation sequencing (NGS) panels. If initial screening by IHC is inconclusive but suspicion remains, these NGS panels would be a logical next step in the diagnostic algorithm. Improved therapies will hopefully follow the increase in identification and accurate diagnosis of these tumors.

Immune checkpoint inhibitors (ICIs) have been identified as a potentially promising treatment approach for SWI/SNF deficient tumors. Response to ICI therapy has been associated with the presence of key predictive biomarkers such as expression status of PD-1 and its ligand PDL-1, high tumor mutation burden, and presence of mismatch repair deficiency. Because this class of tumor remains rare, clinical cases of response to immune checkpoint inhibitors remain anecdotal [14, 15]. In addition to identifying mutations in SWI/SNF genes for diagnostic purposes, due to the large number of genes surveyed in some panels, another useful application is the identification of other targetable mutations that tumors may harbor. In regard to targeted therapy, the inhibitor of the enhancer of zeste homolog 2 (EZH2) when identified by NGS testing has been identified as a targetable mutation for EZH2 inhibition. A phase I clinical trial data using the EZH2 inhibitor tazemetostat in INI1 (SMARCB1)-negative or SMARCA4-negative solid tumors has shown encouraging results, with some patients achieving a complete or partial response and some with prolonged stable disease of greater than 2 years [16].

In conclusion, SMARC-deficient UEC represents a very rare subset of esophageal carcinoma,and thus, research in this disease has been limited. From the available literature, however, it appears that it is more commonly observed in men with age ranges varying. Further research is necessary to better understand this disease and to establish treatment guidelines. While these tumors may not be more aggressive than other esophageal adenocarcinomas when matched stage for stage, it appears from the reported cases that they present at an advanced stage. Additionally, while it may not represent a unique tumor subtype, testing for SMARC deficiency by immunohistochemistry or molecular means may be of clinical utility given the advent of molecularly targeted treatment strategies. In that same vein, HER2 testing would also be advisable for these patients.

## Data Availability

No datasets were generated or analysed during the current study.

## Declarations

### Disclaimer

No new data were generated or analyzed in support of this research, as this is a case report. However, the data underlying the case is available in the article and more information can be shared on reasonable request to the corresponding author.

## References

[1] Sung H, Ferlay J, Siegel RL, Laversanne M, Soerjomataram I, Jemal A, Bray F. Global Cancer Statistics 2020: GLOBOCAN estimates of incidence and mortality worldwide for 36 cancers in 185 countries. CA Cancer J Clin. 2021;71(3):209–49. 10.3322/caac.21660. 33538338 10.3322/caac.21660

[2] Sheikh M, Roshandel G, McCormack V, Malekzadeh R. Current status and future prospects for esophageal cancer. Cancers (Basel). 2023;15(3):765. 10.3390/cancers15030765. 36765722 10.3390/cancers15030765 PMC9913274

[3] Rustgi AK, El-Serag HB. Esophageal carcinoma. N Engl J Med. 2014;371(26):2499–509. 10.1056/NEJMra1314530. 25539106 10.1056/NEJMra1314530

[4] Horton RK, Ahadi M, Gill AJ, Said S, Chen ZE, Bakhshwin A, Nichols M, Goldblum JR, Graham RP. SMARCA4/SMARCA2-deficient carcinoma of the esophagus and gastroesophageal junction. Am J Surg Pathol. 2021;45(3):414–20. 10.1097/PAS.0000000000001599. 33027072 10.1097/PAS.0000000000001599

[5] Ahmed OT, Nam GH, Shui Y, Villavicencio J, Vaziri H. Case series of SMARCA4-deficient undifferentiated esophageal carcinoma. Cureus. 2022;14(10): e30874. 10.7759/cureus.30874. 36457612 10.7759/cureus.30874 PMC9707608

[6] Cui M, Lemmon K, Jin Z, Uboha NV. Esophageal carcinoma with SMARCA4 mutation: unique diagnostic challenges. Pathol Res Pract. 2023;248: 154692. 10.1016/j.prp.2023.154692. 37459679 10.1016/j.prp.2023.154692

[7] Singhi AD, Seethala RR, Nason K, Foxwell TJ, Roche RL, McGrath KM, Levy RM, Luketich JD, Davison JM. Undifferentiated carcinoma of the esophagus: a clinicopathological study of 16 cases. Hum Pathol. 2015;46(3):366–75. 10.1016/j.humpath.2014.11.021. 25582499 10.1016/j.humpath.2014.11.021 PMC4384179

[8] Tian Y, Xu L, Li X, Li H, Zhao M. SMARCA4: current status and future perspectives in non-small-cell lung cancer. Cancer Lett. 2023;554: 216022. 10.1016/j.canlet.2022.216022. 36450331 10.1016/j.canlet.2022.216022

[9] Agaimy A, Daum O, Märkl B, Lichtmannegger I, Michal M, Hartmann A. SWI/SNF Complex-deficient undifferentiated/rhabdoid carcinomas of the gastrointestinal tract: a series of 13 cases highlighting mutually exclusive loss of SMARCA4 and SMARCA2 and frequent co-inactivation of SMARCB1 and SMARCA2. Am J Surg Pathol. 2016;40(4):544–53. 10.1097/PAS.0000000000000554. 26551623 10.1097/PAS.0000000000000554

[10] Karnezis AN, Wang Y, Ramos P, et al. Dual loss of the SWI/SNF complex ATPases SMARCA4/BRG1 and SMARCA2/BRM is highly sensitive and specific for small cell carcinoma of the ovary, hypercalcaemic type. J Pathol. 2016;238(3):389–400. 10.1002/path.4633. 26356327 10.1002/path.4633 PMC4832362

[11] Chang B, Sheng W, Wang L, Zhu X, Tan C, Ni S, Weng W, Huang D, Wang J. SWI/SNF Complex-deficient undifferentiated carcinoma of the gastrointestinal tract: clinicopathologic study of 30 cases with an emphasis on variable morphology, immune features, and the prognostic significance of different SMARCA4 and SMARCA2 subunit deficiencies. Am J Surg Pathol. 2022;46(7):889–906. 10.1097/PAS.0000000000001836. 34812766 10.1097/PAS.0000000000001836

[12] Schallenberg S, Bork J, Essakly A, et al. Loss of the SWI/SNF-ATPase subunit members SMARCF1 (ARID1A), SMARCA2 (BRM), SMARCA4 (BRG1) and SMARCB1 (INI1) in oesophageal adenocarcinoma. BMC Cancer. 2020;20(1):12. 10.1186/s12885-019-6425-3. 31906887 10.1186/s12885-019-6425-3 PMC6945480

[13] Neil AJ, Zhao L, Isidro RA, Srivastava A, Cleary JM, Dong F. SMARCA4 mutations in carcinomas of the esophagus, esophagogastric junction, and stomach. Mod Pathol. 2023;36(6): 100183. 10.1016/j.modpat.2023.100183. 37054973 10.1016/j.modpat.2023.100183

[14] Naito T, Umemura S, Nakamura H, Zenke Y, Udagawa H, Kirita K, Matsumoto S, Yoh K, Niho S, Motoi N, Aokage K, Tsuboi M, Ishii G, Goto K. Successful treatment with nivolumab for SMARCA4-deficient non-small cell lung carcinoma with a high tumor mutation burden: a case report. Thorac Cancer. 2019;10(5):1285–8. 10.1111/1759-7714.13070. 30972962 10.1111/1759-7714.13070 PMC6501032

[15] Iijima Y, Sakakibara R, Ishizuka M, Honda T, Shirai T, Okamoto T, Tateishi T, Sakashita H, Tamaoka M, Takemoto A, Kumaki Y, Ikeda S, Miyazaki Y. Notable response to nivolumab during the treatment of SMARCA4-deficient thoracic sarcoma: a case report. Immunotherapy. 2020;12(8):563–9. 10.2217/imt-2019-0142. 32363992 10.2217/imt-2019-0142

[16] Italiano A, Soria JC, Toulmonde M, Michot JM, Lucchesi C, Varga A, Coindre JM, Blakemore SJ, Clawson A, Suttle B, McDonald AA, Woodruff M, Ribich S, Hedrick E, Keilhack H, Thomson B, Owa T, Copeland RA, Ho PTC, Ribrag V. Tazemetostat, an EZH2 inhibitor, in relapsed or refractory B-cell non-Hodgkin lymphoma and advanced solid tumours: a first-in-human, open-label, phase 1 study. Lancet Oncol. 2018;19(5):649–59. 10.1016/S1470-2045(18)30145-1. 29650362 10.1016/S1470-2045(18)30145-1

